# Tribological Performance of Microhole-Textured Carbide Tool Filled with CaF_2_

**DOI:** 10.3390/ma11091643

**Published:** 2018-09-07

**Authors:** Wenlong Song, Shoujun Wang, Yang Lu, Zixiang Xia

**Affiliations:** 1 Department of Mechanical Engineering, Jining University, Qufu 273155, China; shoujun0531@163.com (S.W.); xiazixiang168@163.com (Z.X.); 2 Department of Mechanical Engineering, Shandong University, Jinan 250061, China

**Keywords:** microhole-textured tool, CaF_2_, micro-EDM, tribological properties

## Abstract

To enhance the friction and wear performance of cemented carbide, textured microholes were machined on micro Electron Discharge Machining (EDM) on the tool rake face, and Calcium Fluoride (CaF_2_) powders were burnished into the microholes. The friction and wear characteristics of the microhole-textured tool filled with CaF_2_ were investigated using sliding friction tests and dry cutting tests. Results exhibited that the working temperature could affect the tribological performance of the microhole-textured tool filled with CaF_2_ due to the temperature-sensitive nature of CaF_2_. There is no obvious lubrication effect for the textured tool filled with CaF_2_ at room temperature, while it was shown to be more effective in improving tribological property at a cutting speed of higher than 100 m/min with a corresponding to cutting temperature of 450 °C. The possible mechanisms for the microhole-textured tool filled with CaF_2_ were discussed and established.

## 1. Introduction

Due to the properties of high surface hardness, good thermal stability, outstanding chemical inertness and excellent wear resistance, cemented carbide has been widely applied in engineering applications [1,2], such as machining tools, engine components, mechanical seal parts and bearing modules. However, without a cutting fluid during the cutting process, the carbide tool will be subjected to more severe friction and wear, leading to the increase of the cutting temperature, abrasive wear and adhesions, and hence the reduction of service life. Accordingly, considerable efforts have been made to improve the cutting performance of carbide tools, i.e., optimal carbide geometries and cutting parameters [1], cryogenic minimum quantity lubrication (MQL) [2], subzero treatment [3,4], thermal treatment [5], and surface coatings such as TiN, TiCN, TiAlN, TiAlSiN and CrSiCN, etc. [6,7,8,9,10,11,12,13]. With superior hardness and chemical stability, the coated cutting tools have significantly promoted the application of carbide inserts.

In recent years, surface textures on sliding surfaces have been utilized to enhance the friction and wear performance, and have been applied in many fields such as bearing rings, engine cylinder blocks and cutting tools [14,15,16,17]. The surface texturing is beneficial to entrap the wear debris, supply lubricant, and enhance the load capacity with fluid lubrication [18,19,20,21,22], which may effectively reduce the friction and wear of the sliding surface. The literature has increasingly been investigating the role of the textured surface in cutting tools. Lei et al. [23] made an array of microholes on the tool surface to carry out the lubrication, and cutting forces were found to be reduced by 10–30% in the turning of hardened steel. Xiong et al. [24] machined surface texturing filled with molybdenum disulfide (MoS_2_) on nickel based composite materials. The sliding tests against alumina balls were carried out with both a textured surface and a non-textured surface. Results showed that the average coefficient of friction and wear rate were decreased for the textured composite filled with MoS_2_ compared to that of the un-textured ones. Kawasegi et al. [25] showed that the texture by a femtosecond laser in the tool-chip contacting zone improved the tribological characteristics owing to the reduction of friction and wear. Deng et al. [26] made micro-textures with various arrays on the carbide tool surface, and MoS_2_ were burnished into the texturing. Results exhibited that the cutting forces, cutting heat and the friction coefficient for the tools with the micro-texture were significantly reduced compared with the smooth ones, and textures with elliptical arrays were superior to those with perpendicular or parallel arrays. They also reported that the textured carbide tools deposited with WS_2_ and TiAlN coatings effectively improved the dry cutting capability [27,28,29].

However, the previous research on surface textures primarily focused on the combination of sulfide additives like MoS_2_ and WS_2_. The sulfides start to oxidize as the operating temperature reaches 450–550 °C, and gradually lose the function of lubrication in higher temperatures [30,31,32,33,34]. Compared with the sulfides, Calcium fluoride (CaF_2_) is a widely utilized solid lubricant at high temperatures. The average friction coefficient of CaF_2_ decreases gradually with the increasing of temperature from about 450 °C, and it still exhibits an excellent lubricating effect at a temperature of 1000 °C [34]. Then CaF_2_ solid lubricant can be used as an addition in the fabrication of ceramic material to improve the friction characteristics. For example, the Al_2_O_3_/TiC based ceramic tool material with the combinations of CaF_2_ powder possessed excellent lubricating performance especially in high-speed turning, owing to elevated temperature [35,36]. Therefore, CaF_2_ was an effective and economical solid lubricant to improve the tribological properties in cutting tools. However, there are few studies on the wear resistance of the textured tool combined with CaF_2_ lubricants [37], and this area therefore still needs a systematic and comprehensive study.

The aim of this paper is to present the friction behavior and wear mechanisms of the microhole-textured carbide tool with a combination of micro-EDM and CaF_2_ lubricants at different temperatures. Sliding tests at lower speeds and cutting tests at higher speeds were implemented using the textured carbide tool and the conventional (untextured) one, while the coefficient of friction, cutting forces, temperature, workpiece surface quality and tool wear was analyzed and compared. Based on the test results, the tribological properties were studied and the corresponding possible reasons for performance improvement were proposed. This study may provide a method of combining surface texturing and CaF_2_ to expand the application of cemented carbide.

## 2. Experimental Procedures

### 2.1. Fabrication of the Microhole-Textured Tool Filled with CaF_2_

**Figure 1:**
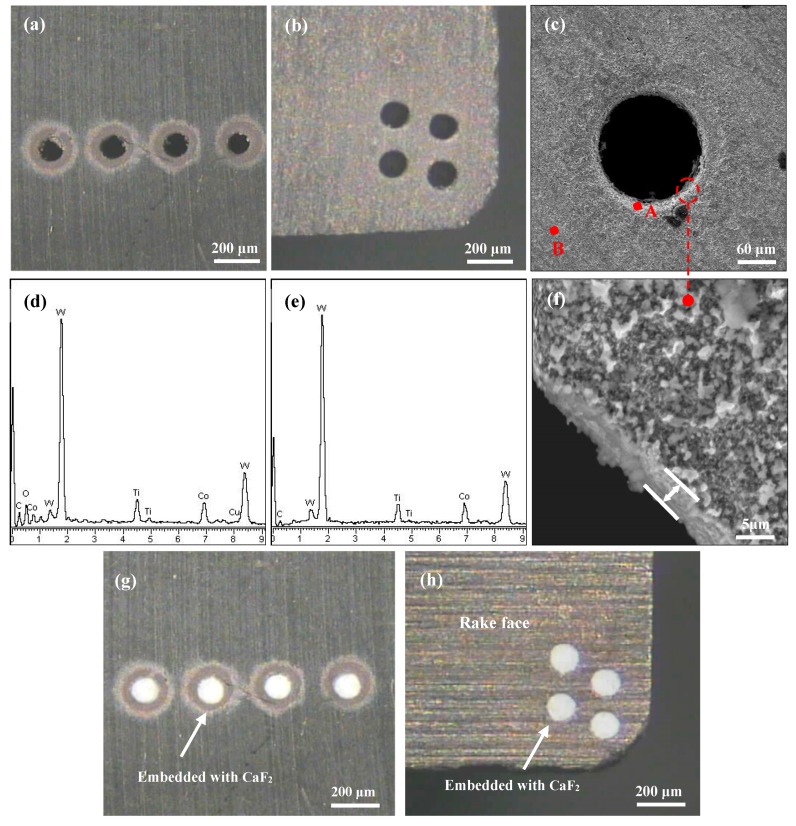
Micrographs of the microholes on the carbide surface: (**a**,**b**) a sample embedded without CaF_2_ for sliding friction test and for cutting test, (**c**) enlarged micrograph corresponding to the micro-hole in (**b**,**d**,**e**) corresponding EDX composition analysis of point A and B in (**c**,**f**) enlarged micrograph corresponding to the micro-hole in (**c**,**g**,**h**) sample embedded with CaF_2_ for the sliding wear test (SC1) and for the cutting test (SCT1).

In this work, carbide insert (WC/TiC/Co) with the size of 16 mm × 16 mm × 4.5 mm was utilized as the test sample. The physical mechanical performance and composition are listed in Table 1. Microholes were then fabricated on the carbide surface using a micro-EDM system (DZW-10, Lunan Machine Tool Co., Ltd., Tengzhou, China). The processing was accomplished with a capacitance of 4.45 nF and voltage of 125 V. Figure 1 shows the scanning electron microscope (SEM) images and corresponding dispersive X-ray (EDX) component analysis on the microholes. To store more lubricants and catch more debris, the average diameter of the microhole was 150 ± 10 μm, the depth was 200 ± 5 μm, and the distance between the micro-holes of the samples for sliding friction test and for cutting tests was 350 μm and 300 μm, respectively. As shown in Figure 1f, the affected layer of the microhole was just 2.5 ± 0.5 μm, which could ignore the influence of EDM on the substrate mechanical properties. The EDX composition analysis was performed before and after fabrication of the micro-EDM indicated in Figure 1d,e. The results obtained are given in Table 2. It indicated that the oxygen and Cu elements were also detected alongside the elements of carbide substrate, and it was clear that these two elements were created and attached to the surface of the microholes during the EDM process.

CaF_2_ powders with an average diameter of 40 nm were manually embedded into the microholes to form microhole-textured tool with combination of CaF_2_. The micrograph of textured carbide filled with CaF_2_ for sliding wear test (SC1) and for cutting tests (SCT1) are shown in Figure 1g,h.

### 2.2. Friction Tests

**Figure 2:**
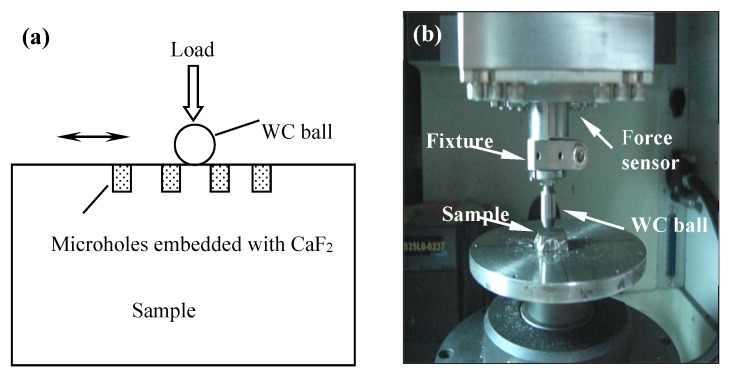
(**a**) Schematic diagram of frictional tester; (**b**) The ball-on-plate tribometer.

Sliding tests of the microhole-textured SC1 sample and conventional smooth one (SC2) were executed using a ball-on-plate tribometer (UMT-2, CETR, Campbell, CA, USA). The schematic diagram of the frictional tester and tribometer are shown in Figure 2a and Figure 2b, respectively. The above sample was a WC carbide ball with a hardness of HRA90 and a diameter of 9.5 mm. The sample below was a WC/TiC/Co carbide sample. The sample below was in a fixed position, and the above ball did linear reciprocal sliding against the counterpart. The tests were implemented with the following parameters: Sliding velocity = 2–10 mm/s, normal force = 10–70 N, stroke sliding = 8 mm and sliding time = 15 min. The worn regions of the specimen were tested using a scanning electron microscope (SEM) and an energy dispersive X-ray (EDX).

### 2.3. Cutting Tests

Cutting experiments were implemented with a CA6140 turning machine (Syms, Shenyang, China) including a conventional fixture with the following parameters: Clearance angle *α*_o_ = 8°, rake angle *γ*_o_ = 8°, side cutting edge angle *k*_r_ = 45°, inclination angle *λ*_s_ = 2°. AISI 1045 quenched steel with a surface hardness of HRC 36–42 was selected as machined material. Cutting tools were utilized with the SCT1 tool and the conventional untextured one (SCT2), and cutting coolant was not applied during the machining experiment. The processing conditions were shown as follows: Cut depth *a*_p_ = 0.2 mm, feed rate *f* = 0.1 mm/r, cutting speed *v* = 60–180 m/min, and cutting time 5 min. Each condition was repeated three times.

**Figure 3:**
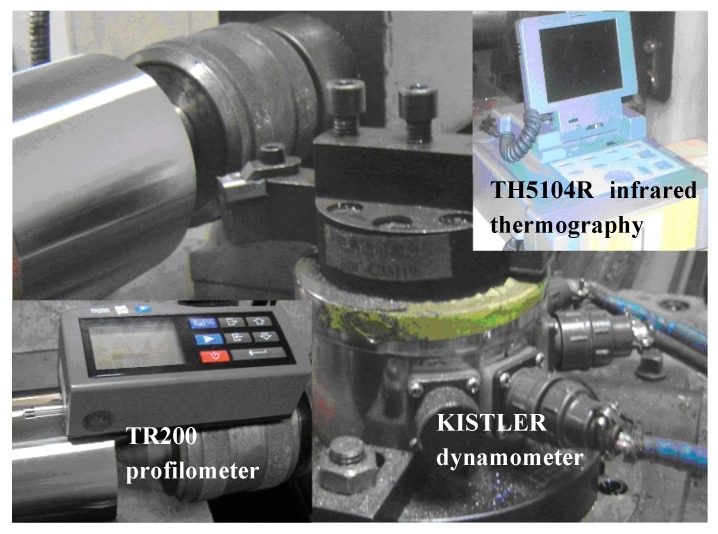
Experimental setup in dry cutting of hardened steel.

Figure 3 presents the setup for the cutting experiment. Cutting forces were evaluated using a KISTLER piezoelectric 9275A quartz dynamometer (Dijia, Chongqing, China). Cutting temperature was measured using a TH5104R infrared thermography (TH5104R, NEC, Tokyo, Japan). The machining quality of workpiece was obtained with a surface profilometer (TR200, SDCH Co., Ltd., Beijing, China) and the sampling length for each test was about 10 mm. The average measurements of the thrice-conducted tests were presented and compared. The micrographs of the worn carbides were observed through SEM (INCA Penta FETXS, Oxford, UK), and the compositions on the corresponding area were analyzed via EDX (D8 ADVANCE, Bruker, Karlsruhe, Germany).

## 3. Results and Discussion

### 3.1. Friction Test and Surface Wear

**Figure 4:**
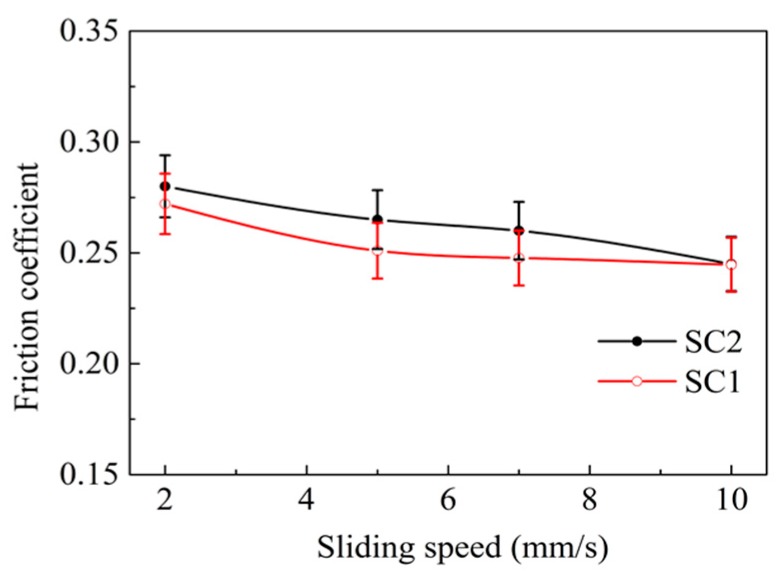
Friction coefficient of the sliding couple at different speeds (load = 50 N).

**Figure 5:**
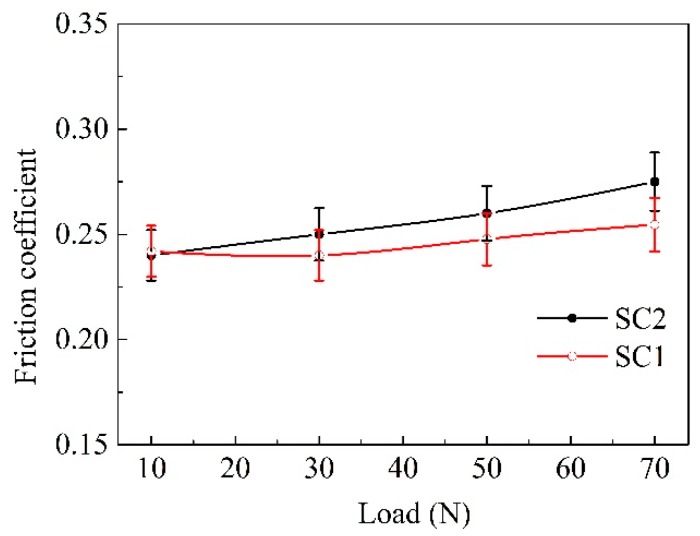
Average friction coefficient of the sliding couple at different loads (speed = 7 mm/s).

Figure 4 and Figure 5 exhibit the friction coefficient of the two kinds of samples in reciprocating sliding wear tests at different sliding speeds and loads. It was evident that there was no marked difference in the friction coefficient between the SC1 and SC2 samples during the test duration. The friction coefficient of the SC2 sample stabilized at about 0.24–0.27, and the SC1 sample possessed a friction coefficient of 0.23–0.26. The average friction coefficient decreased with the increase of the speed, and it increased with the load.

**Figure 6:**
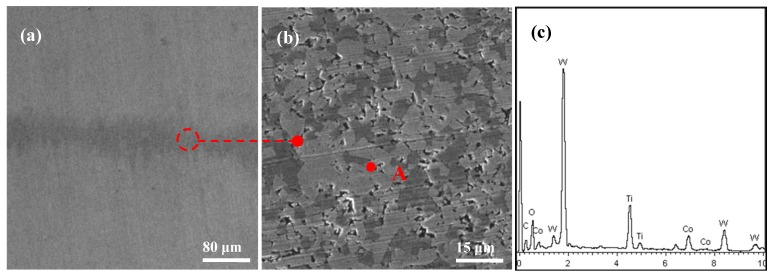
SEM micrographs of the worn surface of the conventional SC2 sample after 15 min sliding friction at the speed of 7 mm/s and load of 50 N: (**a**) SEM micrograph of the wear scar; (**b**) enlarge micrograph corresponding to (**a**); (**c**) EDX composition analysis of point A in (**b**).

Figure 6 shows the SEM micrographs and EDX analysis on the worn track of SC2 sample after 15 min friction test. There was clear abrasive wear on the wear surface, which was characterized as mechanical plowing and scratched appearance. The EDX composition analysis of point A Figure 6c indicated that there were W, Ti and Co elements on the wear face.

**Figure 7:**
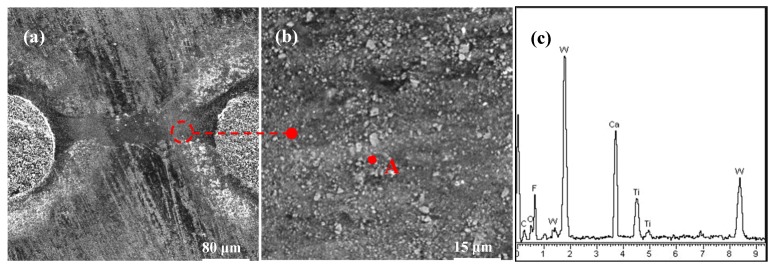
SEM micrographs and EDX composition analysis of the worn surface between two microholes of the SC1 sample after 15 min sliding friction at the speed of 7 mm/s and load of 50 N: (**a**) SEM micrograph of the wear scar; (**b**) enlarge micrograph corresponding to (**a**); (**c**) corresponding EDX composition analysis of point A in (**b**).

Figure 7 exhibits the surface topographies and composition analysis on the wearing area of SC1 sample. No clear abrasive wear can be observed on the friction track between two micro-holes, and large amounts of adhering materials were clearly observed on the sliding track. The EDX analysis in Figure 7c confirmed that the adhesives were CaF_2_ powders, which indicated that CaF_2_ powders were smeared and transferred to the sliding surface from the micro-holes by friction extrusion. Once a thin layer of CaF_2_ was created on the surface, the sliding pairs were separated by the CaF_2_ film, which was beneficial for reducing wear. As a result, the SC1 substrate surface exhibited smaller wear than that of the SC2. However, CaF_2_ powder kept a brittle state at normal temperature [34], and it acted as the abrasive particle in the process of friction, which led to a high coefficient of friction.

### 3.2. Cutting Performance

#### 3.2.1. Cutting Forces

**Figure 8:**
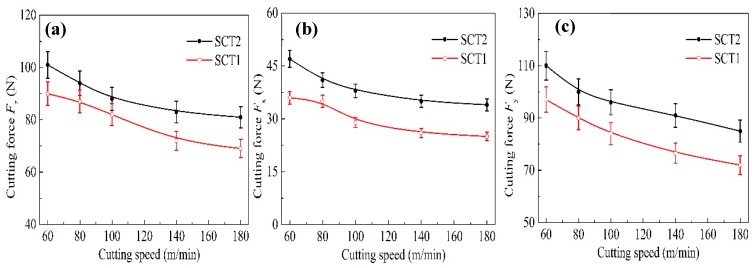
Cutting forces at different speeds with SCT1 and SCT2 in dry cutting of hardened steel (**a**) main force *F*_z_, (**b**) axial thrust force *F*_x_, and (**c**) radial thrust force *F*_y_ (*a*_p_ = 0.2 mm, *f* = 0.1 mm/r, cutting time 5 min).

Figure 8 shows the values of three components of cutting force under different speeds with the SCT1 and SCT2 in machining experiments. From the figure, the turning forces were mainly inversely proportional to the variations of speed. Cutting speed was found to affect the changing rule of force for the SCT1. At a cutting speed of lower than 100 m/min, the three cutting force components for the SCT1 were decreased by about 10–15% compared to the SCT2; while at a cutting speed of higher than 100 m/min, the three cutting force components for the SCT1 were decreased by about 15–25%.

#### 3.2.2. Cutting Temperature

**Figure 9:**
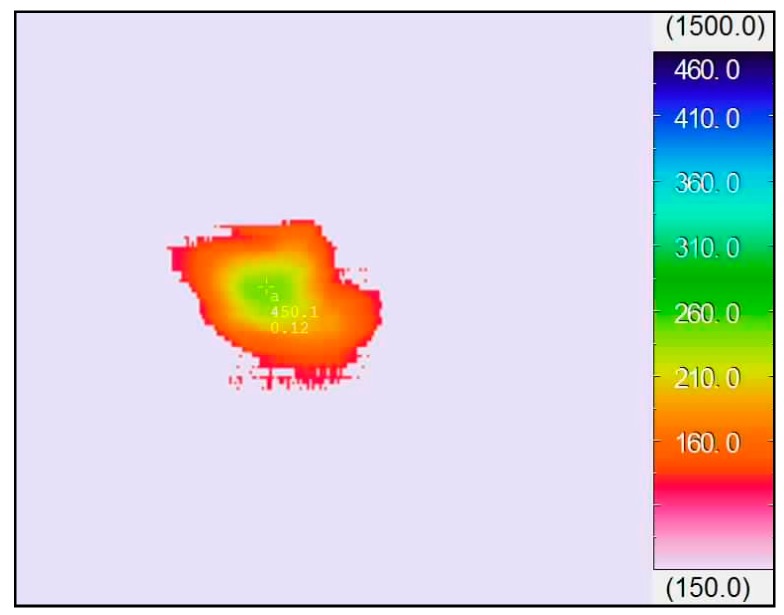
Cutting temperature distribution of chip with SCT1 at speed of 100 m/min in dry cutting hardened steels (*a*_p_ = 0.2 mm, *f* = 0.1 mm/r).

The highest temperature of the chip near the cutting edge was determined by the infrared thermal imaging system in the dry cutting of hardened steels. Figure 9 presents the machining heat energy distribution of chip with SCT1 at turning velocity of 100 m/min, and the highest temperature was about 450 °C under such conditions.

**Figure 10:**
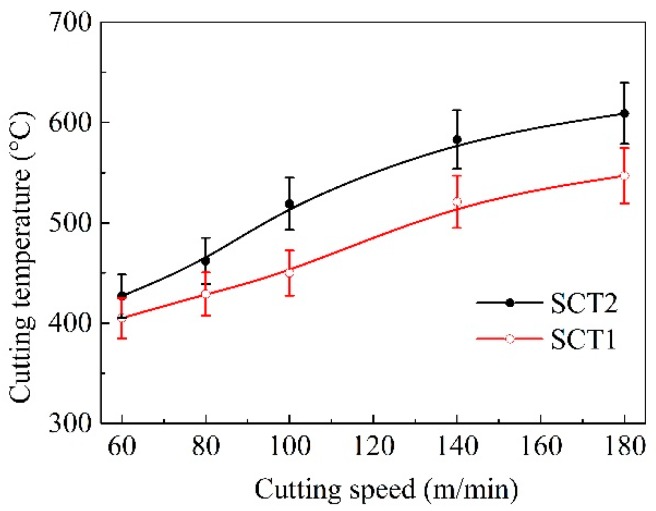
Cutting temperature with SCT1 and SCT2 in dry cutting of hardened steel at different cutting speeds (*a*_p_ = 0.2 mm, *f* = 0.1 mm/r, cutting time 5 min).

The variation temperatures of chip with cutting speed are plotted and shown in Figure 10. It was clear that the temperature with the two tools rose with the turning speed increasing, and it exceeded 450 °C as cutting speed exceeded 100 m/min. The temperature of chip with SCT1 was reduced apparently in comparison with that of the SCT2.

The experimental results also showed that cutting speed affected temperature variation of the SCT1. The temperature of chip with the SCT1 was decreased by 5–10% with speeds lower than 100 m/min; while the cutting temperature was reduced by 10–20% with speeds higher than 100 m/min.

#### 3.2.3. Average Friction Coefficient on the Rake Face

The average coefficient of friction *μ* between the rake face and the chip can be expressed as the formula below [38]:

(1) $$\mu =\tan(\beta )=\tan(\gamma_{o}+\arctan(F_{y}/F_{z}))$$

where *β* is angle of friction, *F*_z_ is primary cutting force, *F*_y_ is radial thrust force and *γ*_o_ is front rake angle. 

**Figure 11:**
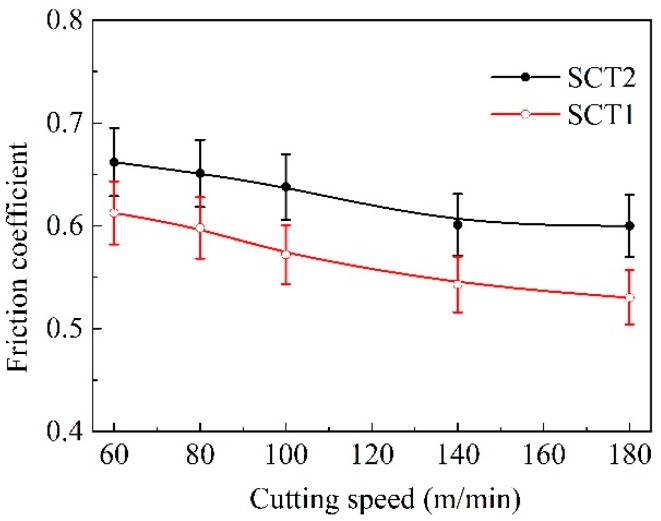
Friction coefficient at the tool-chip interface of SCT1 and SCT2 at different cutting speeds (*a*_p_ = 0.2 mm, *f* = 0.1 mm/r, cutting time 5 min).

Figure 11 presents the variation of the friction coefficient on the cutting tool rake face with machining speed. As indicated in the figure, it could be considered that the SCT1 owned improved surface lubricity on the rake face. Under same machining conditions, the average value of friction coefficient for SCT1 was obviously smaller than that of SCT2 at a cutting speed of higher than 100 m/min; yet there was a relatively small decrease in friction coefficient at a speed of lower than 100 m/min.

#### 3.2.4. Surface Roughness of Machined Workpiece

**Figure 12:**
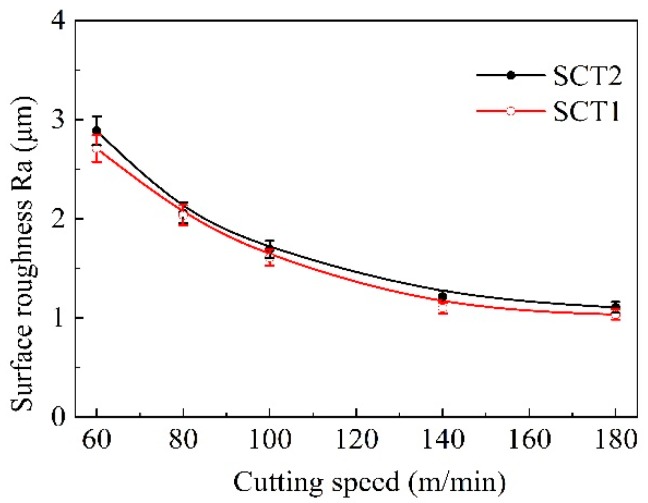
Surface roughness of machined workpiece with SCT1 and SCT2 at different cutting speeds (*a*_p_ = 0.2 mm, *f* = 0.1 mm/r).

Figure 12 indicates the average roughness of the machining surface along with the change of cutting speed. The surface roughness result was an average value of three measurements at different positions. The surface roughness of two kinds of tools exhibited a declining trend with speed increases, and the surface roughness value of SCT1 reduced slightly compared to the value of SCT2.

#### 3.2.5. Wear Properties

**Figure 13:**
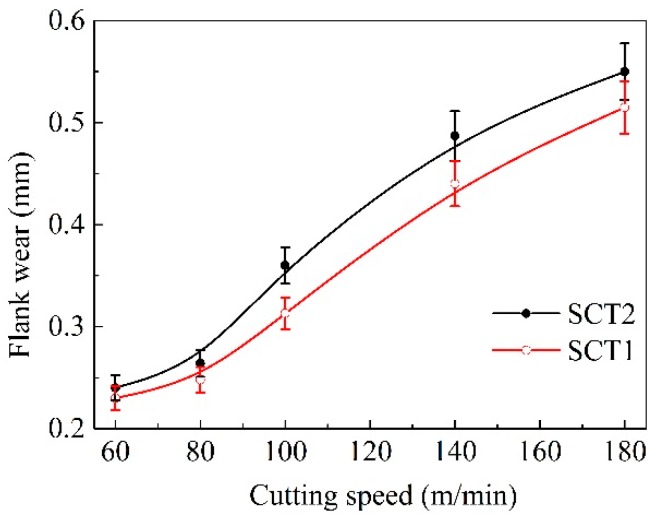
Flank wear of SCT1 and SCT2 tool in dry cutting of hardened steel (*a*_p_ = 0.2 mm, *f* = 0.1 mm/r, cutting time 5 min).

Figure 13 indicates the change of flank wear rate of two tested tools with machining speed. It was evident that the wear of flank was increased with the enhancement of the cutting speed, and the value of flank wear for the SCT1 was lower than the smooth SCT2. This suggests that the microhole-textured tool filled with CaF_2_ was conducive to enhancing the wear resistance of the flank face, especially at cutting speeds of higher than 100 m/min.

**Figure 14:**
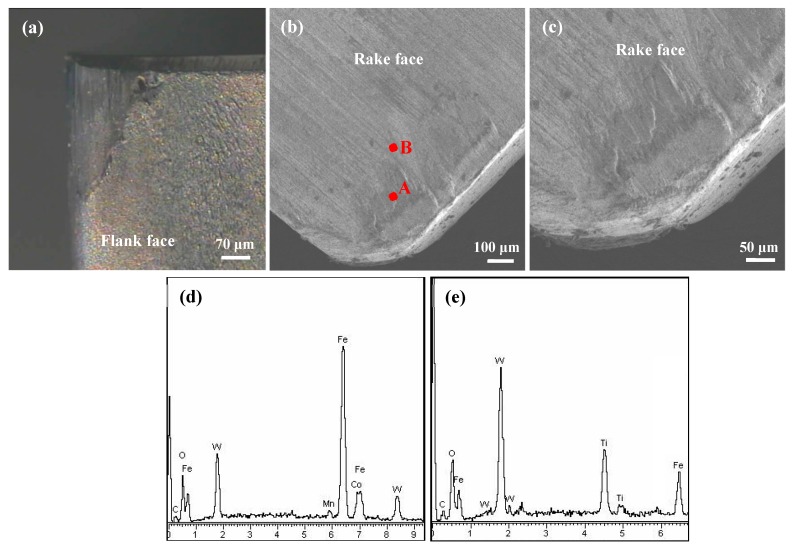
SEM micrographs and EDX component analysis of tool surface of SCT1 after 5 min dry cutting at speed of 100 m/min: (**a**) Micrograph of the worn flank face; (**b**) SEM micrograph of the worn rake face, (**c**) enlarge micrograph corresponding to (**b**,**d**,**e**) corresponding EDX component analysis of point A and B in (**b**).

**Figure 15:**
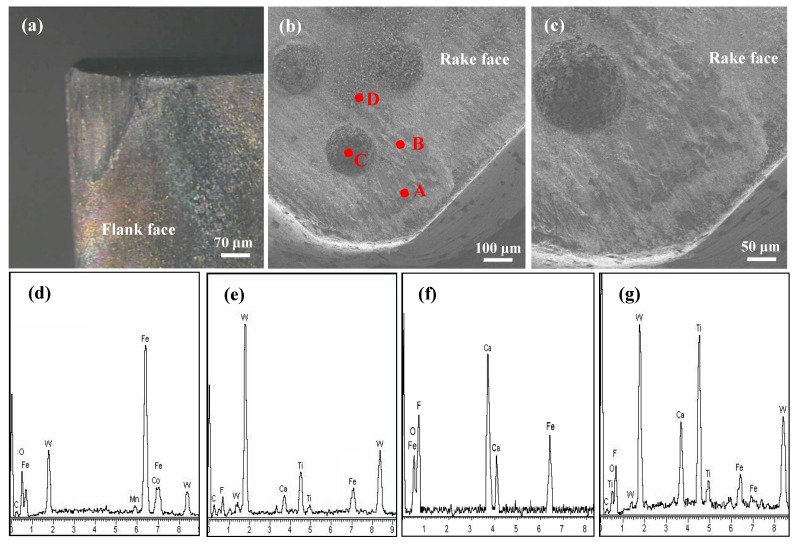
SEM micrographs and EDX component analysis of SCT1surfac after 5 min dry cutting at speed of 100 m/min: (**a**) Micrograph of worn flank face; (**b**) SEM micrograph of worn rake face, (**c**) enlarge micrograph corresponding to (**b**,**d**–**g**) corresponding EDX component analysis of point A, B, C and D in (**b**).

To better evaluate the friction performance and wear mechanism of the tested tools, the wear micrograph and surface component on the worn area for the SCT1 and SCT2 were investigated with SEM and XRD, as indicated in Figure 14 and Figure 15. From Figure 14, significant abrasive wear and evident mechanical ploughs can be found at the flank face (Figure 14a) and rake face (Figure 14b,c) of the SCT2, and clear adhesion material attached to the surface near the tool edge can be observed. The corresponding EDX composition analysis (Figure 14d,e) showed that Fe element was also detected in addition to the elements of WC/Ti/Co carbide tool substrate. This was clearly due to severe friction and chip adhesion that took place on the tool face. The continuous chip friction, attachment and detachment of chip to the tool surface may exacerbate the wear of the flank face, rake face and cutting edge.

As shown in Figure 15, there were also many plows and types of adhering material, but the flank wear, rake wear and edge wear of the SCT1 were mild in contrast to the SCT2. The corresponding surface composition measurements on the wear face are shown in Figure 15d–g. It can be determined that there were iron materials of workpiece in the microholes (Figure 15f) and on the tool face (Figure 15d,e,g), and the CaF_2_ lubricants were dragged out from the microholes and applied on the tool face in cutting process (Figure 15e,g). The results identified that a continuous and/or discontinuous CaF_2_ lubricating layer had been produced on the friction track of the SCT1, which was conducive to reducing the wear of cutting edge and tool surface. In addition, the microholes were beneficial to entrap the wear debris and in doing so slow down abrasion and adhesion of the workpiece on the tool face, and supply more CaF_2_ lubricants.

As shown in the figures above, it could be considered that the main wear mechanisms of rake face for the samples were abrasive and adhesive wear, and abrasive wear was the main wear mechanism of the flank face.

## 4. Discussion

The test results showed that the microhole-textured tool filled with CaF_2_ was ideal for the enhancement of tribological performance. The mechanisms responsible for the improvement of tribological properties of the SCT1 are discussed next.

### 4.1. Cutting Forces

During practical machining, the average frictional force *F*_f_ on tool rake face can be expressed as [39]:

(2) $$F_{f}=a_{w}l_{f}{\bar{\tau }}_{c}=a_{w}l_{f}(k\tau_{c}+(1-k)\tau_{f})$$

where *F*_f_ is frictional force on tool surface, ${\bar{\tau }}_{c}$ is the average shear strength of tool surface, *l*_f_ is the superficial tool-chip contact length, *a*_w_ is the width of cutting, *k* is the ratio of effective contact length to superficial contact length, *τ*_c_ is the shear strength of the machined workpiece, and *τ*_f_ is the shear strength of lubricating film on the tool surface.

**Figure 16:**
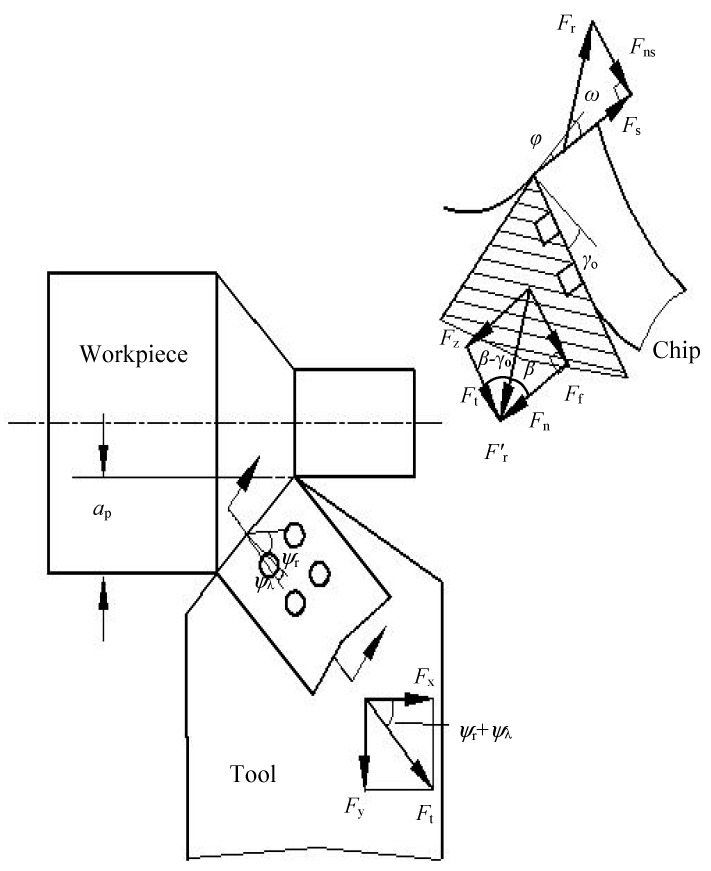
Simplified model of oblique cutting.

Then, the three force components of similar oblique cutting shown in Figure 16 can be determined as follows [40,41]:

(3) $$F_{z}=F_{r}\cos\left(\beta -\gamma_{o}\right)=\frac{F_{f}}{\sin\beta }\cos\left(\beta -\gamma_{o}\right)=a_{w}l_{f}(k\tau_{c}+(1-k)\tau_{f})\left(\sin\gamma_{o}+\frac{\cos\gamma_{o}}{\tan\beta }\right)$$

(4) $$F_{x}=F_{r}\sin\left(\beta -\gamma_{o}\right)\cos(\psi_{r}+\psi_{\lambda })=a_{w}l_{f}(k\tau_{c}+(1-k)\tau_{f})\left(\cos\gamma_{o}-\frac{\sin\gamma_{o}}{\tan\beta }\right)\cos(\psi_{r}+\psi_{\lambda })$$

(5) $$F_{y}=F_{r}\sin\left(\beta -\gamma_{o}\right)\sin(\psi_{r}+\psi_{\lambda })=a_{w}l_{f}(k\tau_{c}+(1-k)\tau_{f})\left(\cos\gamma_{o}-\frac{\sin\gamma_{o}}{\tan\beta }\right)\sin(\psi_{r}+\psi_{\lambda })$$

where *F*_x_ is the axial force, *F*_r_ is the cutting force component of the shear plane, $\psi_{r}$ is the approach angle, ϕ is the shear angle, and $\psi_{\lambda }$ is the angle of chip flow.

**Figure 17:**
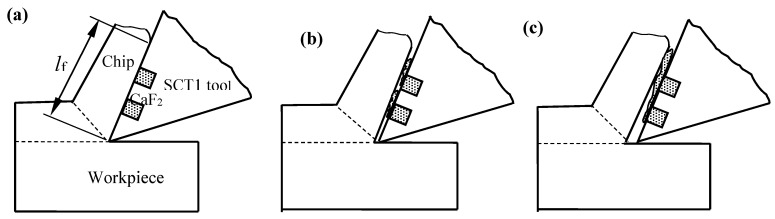
Lubricating model of SCT1 tool in machining process: (**a**) cutting beginning; (**b**) CaF_2_ solid lubricant piled out; (**c**) film forming.

Equations (2)–(5) demonstrate that the three cutting force components have a variation of direct proportion with the average shear strength ${\bar{\tau }}_{c}$ and tool–chip contact length *l*_f_. The thermal expansion coefficient of CaF_2_ (18.38 × 10^−6^/K) is significantly larger than that of the tool substrate (6.21 × 10^−6^/K). The CaF_2_ powders could be dragged out from the microholes because of high cutting temperature and chip friction, and attach to the rake face unevenly. Then a continuous and/or discontinuous CaF_2_ lubricating layer may form on the tool surface, which is consistent with the results obtained in Figure 15. The sliding condition between chip and tool is converted from dry friction to boundary friction. That is, the tool substrate endures the load, the fiction occurs on the lubricating film, and self-lubricity is achieved. The lubricating model of the SCT1 in machining process is shown in Figure 17. Supposing that ratio *k* is 0.8, the ratio of contact length covered with CaF_2_ layer is about 0.2. Owing to the much smaller shear stress of CaF_2_, the average shear strength ${\bar{\tau }}_{c}$ of the SCT1 will be reduced by about 20 percent. Then the three cutting force components will be decreased by about 20% based on Equations (3)–(5). At the same time, forming a CaF_2_ lubricating layer between tool-chip can lead to a reduction of chip distortion and angle of friction [40], which is beneficial to a further decrease of cutting forces. Thus, the formation of CaF_2_ lubricating layer on the tool surface can efficiently decrease the cutting forces.

Furthermore, the microholes on the tool surface can reduce the tool-chip contact length *l*_f_ as indicated in Figure 17, and the effective contact length *l*_a_ can be calculated as:

*l*_a_ = *l*_f_ − *nd* (6)

where *d* is the microhole diameter and *n* is the microhole quantity in the effective contact area.

As the initial length is 0.8 mm, and there are two microholes of 0.15 mm in diameter at the contact area, the effective length *l*_a_ will change to 0.5 mm (Figure 16), and the three cutting force components will be decreased by about 37.5% without loss in mechanical properties according to Equations (3)–(5), due to the decrease of the tool-chip contact area.

### 4.2. Cutting Temperature

**Figure 18:**
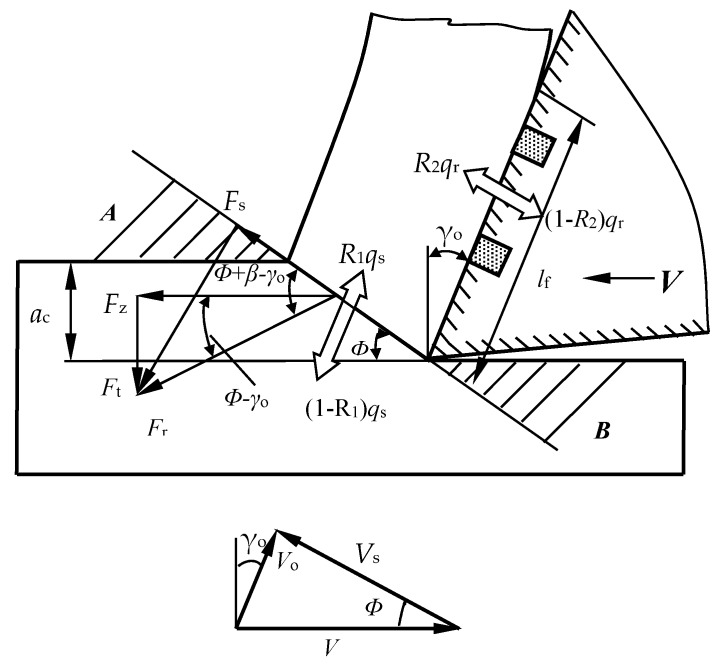
Schematic diagram of the heat distribution during cutting process.

The heat produced in the metal machining process mainly consists of three components [40]: The elastic-plastic deformation of chip on the shear plane, the friction between tool rake face and chip, and the friction between tool flank face and processed surface of workpiece. As a rule, the friction of tool flank has little influence and can be neglected, and the cutting heat can be simplified in calculation. A schematic diagram of the cutting heat distribution is presented in Figure 18, and the average temperature of cutting tool (${\bar{\theta }}_{\mathrm{tt}}$) and chip (${\bar{\theta }}_{t}$) can be expressed as [40,41]:

(7) $${\bar{\theta }}_{t}={\bar{\theta }}_{s}+{\bar{\theta }}_{f}=\theta_{0}+\frac{R_{1}\tau_{s}\cos\gamma_{o}}{c_{1}\rho_{1}(\sin(2\phi -\gamma_{o})+\sin\gamma_{o})}+0.7524R_{2}{\bar{\tau }}_{c}\sqrt{\frac{k_{2}va_{w}l_{f}}{c_{2}\rho_{2}\xi }}$$

(8) $${\bar{\theta }}_{\mathrm{tt}}={\bar{\theta }}_{s}+{\bar{\theta }}_{\mathrm{ft}}=\theta_{0}+\frac{R_{1}\tau_{s}\cos\gamma_{o}}{c_{1}\rho_{1}(\sin(2\phi -\gamma_{o})+\sin\gamma_{o})}+0.7524(1-R_{2}){\bar{\tau }}_{c}\sqrt{\frac{k_{2}va_{w}l_{f}}{c_{2}\rho_{2}\xi }}$$

where ${\bar{\theta }}_{s}$ is shear plane temperature of the chip, ${\bar{\theta }}_{\mathrm{ft}}$ and ${\bar{\theta }}_{f}$ is the temperature increase of the cutting tool and chip, respectively, caused by friction of the tool and chip, *R*_1_ is the proportion between the heat of chip and the whole heat caused by chip deformation, *c*_1_ is heat capacity of the chip as the temperature is ($\theta_{0}$ + ${\bar{\theta }}_{s}$)/2, *ρ*_1_ is the workpiece density,ϕ is the shearing angle, *τ*_s_ is the workpiece shear strength, *ξ* is the chip deformation coefficient, *θ*_0_ is ambient temperature, *R*_2_ is the proportion between the heat of chip and the whole heat produced by severe tool-chip friction, *k*_2_ is thermal diffusivity coefficient of chip as temperature is (2${\bar{\theta }}_{s}$ + ${\bar{\theta }}_{f}$)/2, *c*_2_ and *ρ*_2_ is the heat capacity and density of the chip respectively as the temperature is (2${\bar{\theta }}_{s}$ + ${\bar{\theta }}_{f}$)/2.

According to Equations (7) and (8), the average cutting temperature of the cutting tool and chip are both positively correlated with the shear strength ${\bar{\tau }}_{c}$ and tool-chip contact length *l*_f_. Owing to the reduced shear strength and contact area, the cutting temperature of the SCT1 goes down in comparison with that of SCT2; meanwhile, from Lee and Shaffer shear angle formula [38], the decreased friction angle *β* can bring about the increase of shear angle ϕ, which is also propitious for the decrease of chip temperature on the shear plane. This is well consistent with the cutting temperature results shown in Figure 10.

Furthermore, the CaF_2_ solid lubricant had a smaller thermal conductivity (9.17 W/(m·K)) compared to the carbide insert (33.47 W/(m·K)). Once a continuous and/or discontinuous CaF_2_ film is created on the carbide surface, the thin film could act as a thermal barrier to prevent the heat transfer to the carbide substrate, which is propitious to further lower tool temperature and tool wear.

### 4.3. Average Friction Coefficient at The Sliding Interface

The average friction coefficient between two elastic contact surfaces in sliding can be represented as [39]:

(9) $$\mu =\tan\beta =\frac{F_{f}}{P}=\frac{{\bar{\tau }}_{c}A_{r}}{\sigma_{b}A_{r}}=\frac{{\bar{\tau }}_{c}}{\sigma_{b}}=\frac{k\tau_{c}+(1-k)\tau_{f}}{\sigma_{b}}$$

where *P* is the normal load, *A*_r_ is the actual contact area, and *σ*_b_ is the compressive yield limit of tool substrate materials.

It can be seen that a CaF_2_ lubricating layer attached to the tool surface conduces to lower friction coefficient for the SCT1 by Equation (9); meanwhile, the microholes on the rake face by reasonable design can supply more lubricant Figure 15e,g and entrap more wear debris of chip Figure 15c, which are conducive to the reduction of friction coefficient. This was in accordance with the variation of the friction coefficient obtained in Figure 11.

Service temperature had an obvious influence on the tribological performance of the microhole-textured carbide filled with CaF_2_. This is because the CaF_2_ solid lubricant is a wonderful lubricating material suitable for high temperatures, it can effectively carry out lubrication in the range of 450–700 °C, and still maintains good lubricating performance even at a temperature of 1000 °C [34]. However, if the working temperature drops below 400–450 °C, CaF_2_ begins to transit from ductile to brittle mode, and the average coefficient of friction increases gradually for lower temperatures. Therefore, the textured carbide embedded with CaF_2_ powders can more efficiently implement lubrication at a higher cutting speed with corresponding to a higher temperature (Figure 8, Figure 9, Figure 10, Figure 11, Figure 12, Figure 13, Figure 14 and Figure 15), and result in improved cutting performance. But at a lower cutting speed, the textured carbide exhibits a relatively poor lubricating performance, and even loses the lubricating effect at room temperature, which has been confirmed by the sliding tests and cutting tests as shown in Figure 4, Figure 5, Figure 6, Figure 7, Figure 8, Figure 9, Figure 10, Figure 11, Figure 12 and Figure 13.

Future investigations will be carried out on the lifetime of textured tools under different test conditions (speed, load, temperature, etc.), and will seek to determine what is the cutting performance without a lubricant supply after a long period of service.

## 5. Conclusions

The study presented the tribological properties of a microhole-textured carbide tool filled with CaF_2_. The friction performance and antiwear mechanism of the textured carbide tool during sliding friction tests and dry machining tests were investigated and studied. The main conclusions are as follows:

1. There was no significant change in the friction coefficient of the conventional microhole-textured carbide filled with CaF_2_ (SC1) and an conventional one (SC2) in sliding tests with WC ball.
2. Compared with the untextured carbide tool (SCT2), the microhole-textured carbide tool filled with CaF_2_ (SCT1) was effective in promoting machining performance. The tool rake face revealed adhesion and abrasive wear, and flank face indicated severe abrasive wear.
3. Service temperature was found to affect the tribological performance of the textured carbide, which was probably due to the sensitivity of CaF_2_ solid lubricant to the cutting temperature. At machining speeds higher than 100 m/min, corresponding to temperature of 450 °C, the textured carbide improved the tribological performance compared to the untextured carbide; while at machining speeds lower than 100 m/min, the tribological properties of the textured carbide were only slightly improved in comparison with the smooth one, and it lost the lubrication effect at room temperature.
4. The reasons of performance improvement for the textured tool were as follows: Owing to high cutting heat and friction, CaF_2_ powders may be drawn out of the microhole textures, adhere to the tool surface and create an uneven CaF_2_ layer on the rake face, which is propitious to reducing cutting forces, cutting temperature, friction coefficient and tool wear. On the other hand, the microhole textures at the tool face could lower the tool-chip contact length and entrap workpiece debris, which is beneficial to increasing machining performance.

## Figures and Tables

**Table 1:** Properties of the cemented carbide material.

Composition (wt. %)	Density (g/cm^3^)	Hardness (GPa)	Flexural Strength (MPa)	Young’s Modulus (GPa)	Thermal Expansion Coefficient (10^−6^/K)	Poisson’s Ratio
WC + 15%TiC + 6%Co	11.5	15.5	1130.0	510	6.51	0.25

**Table 2:** Element compositions analysis of the cemented carbide before and after EDM.

Element Content	Before EDM (wt. %)	After EDM (wt. %)
C	11.41	20.97
O	0	10.6
Ti	5.57	5.31
Co	8.18	5.84
Cu	0	0.37
W	74.84	56.91
Total	100%	100%
